# Renal Tissue Expression of BAFF and BAFF Receptors Is Associated with Proliferative Lupus Nephritis

**DOI:** 10.3390/jcm12010071

**Published:** 2022-12-22

**Authors:** Miguel Marín-Rosales, Claudia Azucena Palafox-Sánchez, Ramón Antonio Franco-Topete, Francisco Josué Carrillo-Ballesteros, Alvaro Cruz, Diana Celeste Salazar-Camarena, José Francisco Muñoz-Valle, Francisco Ramos-Solano

**Affiliations:** 1 Departamento de Reumatología, Hospital General de Occidente, Secretaría de Salud Jalisco, Guadalajara 45170, Mexico; 2 Grupo de Inmunología Molecular, Centro Universitario de Ciencias de la Salud, Universidad de Guadalajara, Guadalajara 44340, Mexico; 3 Instituto de Investigación en Ciencias Biomédicas (IICB), Centro Universitario de Ciencias de la Salud, Universidad de Guadalajara, Guadalajara 44340, Mexico; 4 Departamento de Microbiología y Patología, Centro Universitario de Ciencias de la Salud, Universidad de Guadalajara, Guadalajara 44340, Mexico; 5 Departamento de Farmacobiología, Centro Universitario de Ciencias Exactas e Ingenierías, Universidad de Guadalajara, Guadalajara 44430, Mexico

**Keywords:** systemic lupus erythematosus, proliferative lupus nephritis, BAFF system expression

## Abstract

Background: The B-cell activating factor (BAFF) controls the maturation and survival of B cells. An imbalance in this cytokine has been associated with systemic autoimmunity in SLE and lupus nephritis (LN). However, few investigations have evaluated the tissular expression of BAFF in LN. This study aimed to associate BAFF system expression at the tissular level with the proliferative LN classes. Methods: The analysis included eighteen kidney tissues, with sixteen LN (class III = 5, class IV = 6, class III/IV+V = 4, and class V = 1), and two controls. The tissular expression was evaluated with an immunochemistry assay. A Cytation5 imaging reader and ImageJ software were used to analyze the quantitative expression. A *p*-value < 0.05 was considered significant. Results: The expressions of BAFF, A proliferation-inducing ligand (APRIL), and their receptors were observed in glomerular, tubular, and interstitial zones, with BAFF being the most strongly expressed in the overall analysis. BAFF-Receptor (BR3), transmembrane activator and CALM interactor (TACI), and B-Cell maturation antigen (BCMA) displayed higher expressions in LN class IV in all zones analyzed (*p* < 0.05). Additionally, a positive correlation was found between APRIL, TACI, and BCMA at the glomerular level; BCMA and APRIL in the interstitial zone; and BR3, TACI, and BCMA in the tubule (*p* < 0.05). Conclusions: The expression of BAFF and BAFF receptors is mainly associated with LN class IV, emphasizing the participation of these receptors as an essential pathogenic factor in kidney involvement in SLE patients.

## 1. Introduction

Lupus nephritis (LN) is the most frequent life-threatening clinical domain of systemic lupus erythematosus (SLE); renal involvement affects 30–70% of patients and shows high frequency and severity in Black and Latin-American Hispanic populations [1,2]. LN diagnosis is sustained based on clinical features and an evaluation of conventional biomarkers such as low complement serum concentration, high anti-dsDNA antibodies, and urinary findings [3,4]. However, kidney biopsy is the diagnostic gold standard and guides LN treatment, according to the 2003 International Society of Nephrology (ISN)/Renal Pathology Society (RPS) classification [5,6].

B-cell ontogeny is controlled through the BAFF system; this system is integrated by B-cell activating factor (BAFF), a proliferation-inducing ligand (APRIL), and their receptors, namely BAFF-Receptor (BR3), transmembrane activator and CALM interactor (TACI), and B-Cell maturation antigen (BCMA). BAFF and APRIL belong to the tumor necrosis factor (TNF) family and promote B-cell activation, proliferation, maturation, and survival through BR3, TACI, and BCMA receptors [7,8,9,10,11].

An imbalance in BAFF, APRIL, or their receptors in both murine models and humans has been associated with the development of autoimmune diseases, including SLE, Sjögren’s Syndrome, and rheumatoid arthritis [11,12,13,14,15,16,17,18]. Regarding SLE, the serum concentration of BAFF and APRIL has been reported to be higher in SLE patients; these ligands were previously associated with the disease activity index and autoantibody levels and can predict a flare [13,14,19,20,21,22,23,24,25]. On the other hand, the soluble BCMA serum concentration was found to have a higher concentration and was correlated with the activity index in SLE patients [25]. Regarding the BAFF receptors, BR3, TACI, and BCMA were identified in CD3 T cells in SLE patients, and their expression varies according to SLE disease activity [21,23,25].

Given the growing need for diagnostic tools for LN, multiple new biomarkers have been associated with this domain [26], and BAFF and APRIL have been linked with renal activity in Mexican SLE patients [22,23]. Additionally, BAFF and their receptors were analyzed in situ in kidney tissues of LN patients, showing differential pattern expression according to LN classes [27]. However, the analysis did not include APRIL. Based on the association of the BAFF system with renal involvement, the poor renal prognosis for proliferative LN in Latin American SLE patients, and the small number of validated renal biomarkers, this study evaluated the renal tissue expression of BAFF, APRIL, BR3, TACI, and BCMA in patients with proliferative LN.

## 2. Materials and Methods

### 2.1. Study Tissues

A retrospective and descriptive study were conducted. This study included sixteen kidney tissues of LN patients classified as class III, IV, V, or V/III-IV according to the 2003 ISN/RPS classification [5]. All LN patients met the 2012 Systemic Lupus International Collaborating Clinics classification criteria for SLE [28] and were recruited at the rheumatology department of Hospital General de Occidente. Additionally, two kidney incisional biopsies without autoimmunity histopathological features were used as controls. All patients provided written informed consent according to the 2013 Declaration of Helsinki and actual national guidelines of the Health Ministry. The ethics and research committees of the Hospital General de Occidente, Jalisco, Mexico, approved the study under the number CI-561/18.

### 2.2. Immunohistochemical Staining

Kidney biopsies were fixed in 4% paraformaldehyde and embedded in paraffin using a tissue processor (TP1020; Leica Biosystems, Wetzlar, Germany). Tissues were sectioned at 5 µm, mounted on electrocharged slides, and deparaffinized at 60 °C for 30 min in a Dako Hybridizer (Dako Colorado Inc. Collins, CO, USA). Posteriorly, the tissues were rehydrated by immersion in xylene using graded ethanol dilutions followed by distilled water. According to Carrillo-Ballesteros et al., the immunohistochemical assay was standardized in amygdaline tissue [29]. Briefly, after rehydration of the tissue, epitope retrieval was performed with the Dako PT Link system (Dako Colorado Inc. Collins, CO, USA) at 90 degrees for 30 min, and the slides were submerged into the Dako PT Link cameras with a 1 mM EDTA buffer (pH = 9) for BAFF, APRIL, and BR3, as well as a 10 mM sodium citrate buffer (pH = 6) for TACI and BCMA. Later, all slides were cooled, and a 3% hydrogen peroxide—10% methanol solution was added to achieve an endogenous peroxidase blockade. Posteriorly, slides were incubated for 30 min with the following primary antibodies: rat monoclonal antibody to BAFF (Abcam, cat. no. ab16081, dilution 1:100), rabbit polyclonal antibody to APRIL (Abcam, cat. no. ab189263, dilution 1:50), mouse monoclonal antibody to BR3 (Abcam, cat. no. ab16232, dilution 1:250), rabbit polyclonal antibody to TACI (Abcam, cat. no. ab79023, dilution 1:100), and rabbit polyclonal antibody to BCMA (Abcam, cat. no. ab5972, dilution 1:100). Following incubation with primary antibodies and buffer washing, the slides were incubated for 10 min with the universal post-primary antibody included in the BOND polymer refine detection system (Leica Biosystems, cat. no. DS9800), and diaminobenzidine (DAB) was employed for detection until the development of a red-brown color. Finally, slides were counterstained with Harris hematoxylin and reviewed under a microscope by two experienced pathologists.

### 2.3. Immunohistochemical Image Processing and Statistical Analysis

After slide tissue staining, three renal structures (glomerulus, tubule, and interstitial) were photographed in triplicate using a BioTek Citation|5 Cell Imaging Multimode Reader (Santa Clara, CA, USA) with 10× and 20× magnification. Later, the ImageJ v1.51j8 and DAB deconvolution plugin software (National Institute of Health, Bethesda, MD, USA. https://imagej.nih.gov/ij/, accessed on 15 January 2021) was used to analyze the photos and obtain the immunoreaction score. In the DAB layer, the pixel intensity value was represented in a range of 0–255 (the darkest shade and lightest shade represent 0 and 255, respectively), according to Chatterjee et al. [30]. The mean intensity default threshold was set in the ImageJ software under the “Image” menu using the “measure” tool from the “Analyze” menu. Later, the percentage of positive pixels was determined in the selected areas. Descriptive analysis included the median, interquartile range (IQR), and frequencies. Additionally, Fisher, Chi-square, Mann–Whitney U, Kruskal–Wallis and Dunn’s post hoc, and Spearman correlation tests were used as appropriate. A *p*-value < 0.05 was considered significant.

## 3. Results

### 3.1. Histopathological Features of LN

Eighteen kidney tissues were included, including sixteen percutaneous biopsies of patients with LN, and two incisional biopsies were obtained by necropsy without histopathological features of autoimmunity. Thirteen (81%) LN cases were women, class IV represented 38% (6/16) of the cases, class III and class V+III/IV were 31% (5/16) and 25% (4/16) of the cases, respectively, and only 6% (1/16) were classified as class V. The wire-loop lesions and total renal activity index were associated with proliferative diffuse LN (*p* < 0.05). Table 1 shows all histopathological findings.

### 3.2. Renal BAFF System Expression and Association with LN Classes

**Figure 1:**
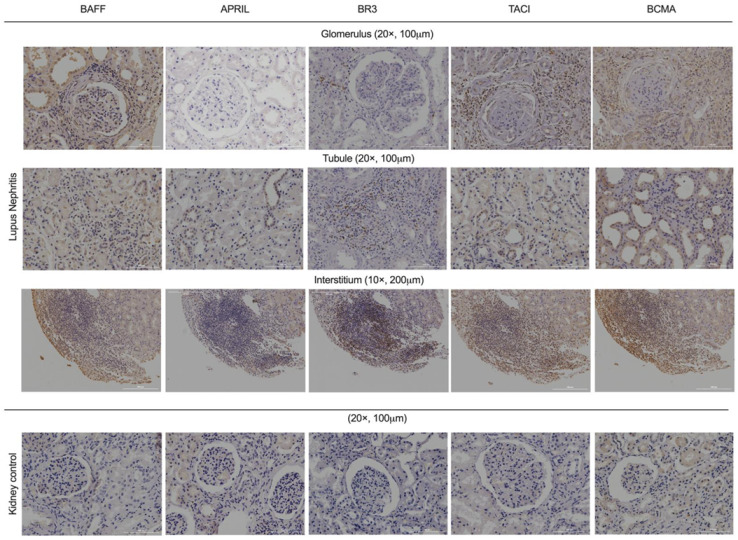
BAFF system expression in lupus nephritis tissues and kidney controls. Glomerular, tubular, and interstitial zones have a positive stain directed to BAFF system members. BR3 and TACI show more stains in the interstitial infiltration. Thus, APRIL was the lowest. The inflammatory cells in the interstitium could simulate an ectopic germinal center. Kidney control tissues did not show expression of BAFF, APRIL, or their receptors. BAFF: B-cells activating factor, APRIL: A proliferation-inducing ligand, BR3: BAFF receptor, TACI: Transmembrane activator and CALM interactor, BCMA: B-cell maturation antigen.

The LN tissues showed BAFF system expression at the glomerular, tubule, and interstitial levels. BAFF and BCMA were expressed in the glomerular epithelium membrane of the glomerulus, and TACI presented expression in glomerular resident cells. BAFF, APRIL, TACI, and BCMA were expressed at the tubular level, mainly in the cytoplasm of the tubular epithelium. Additionally, BR3, TACI, and BCMA demonstrated expression in inflammatory cells at an interstitial level. The control kidney tissues showed low BAFF expression in the tubular system, and APRIL, BR3, TACI, and BCMA were not expressed. Figure 1 shows BAFF system expression in tissues with LN and renal controls.

**Figure 2:**
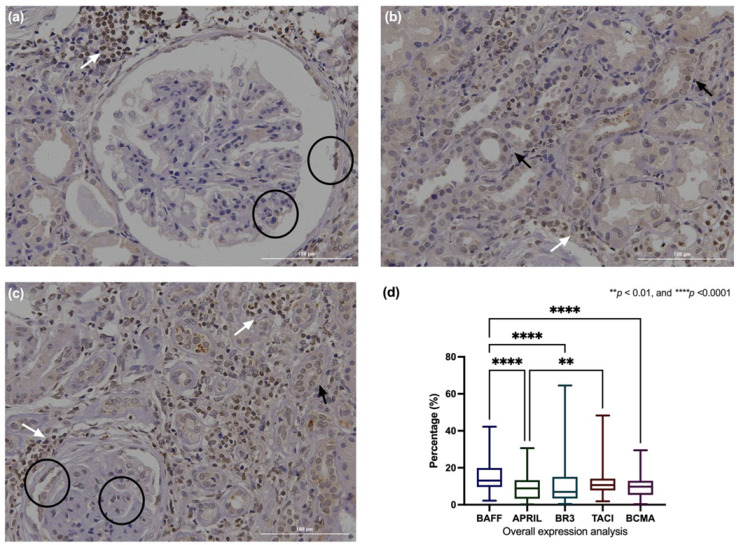
Overall analysis of BAFF system expression in LN. BAFF system expression according to glomerular, tubular, and interstitial zones is illustrated in (**a**–**c**). Black circles, black arrows, and white arrows indicate glomerular, tubular, and interstitial BAFF system expression, respectively. The overall expression analysis indicated a higher expression of BAFF and TACI than APRIL. Additionally, BAFF displays a higher expression of BR3 and BCMA (**d**). The *p*-value was obtained through Kruskal–Wallis and post hoc tests. Data are shown as the median with IQR. BAFF: B-cells activating factor, APRIL: A proliferation-inducing ligand, BR3: BAFF receptor, TACI: Transmembrane activator and CALM interactor, BCMA: B-cell maturation antigen, I IQR: Interquartile range.

Based on the previous findings, ImageJ software was used to perform a quantitative expression analysis. For the LN tissues, the overall analysis indicated a higher percentage of BAFF expression [13.07% (IQR 9.62–19.91%)] than APRIL [8.86 (IQR 3.28–12.95%)], BR3 [6.93% (IQR 3.40–15.07%)], and BCMA [9.80 (IQR 5.32–12.95%)], with statistical significance (*p* < 0.05). Additionally, TACI [10.66% (IQR 7.74%–14.11%)] had higher expression than APRIL (*p* < 0.05) (see Figure 2d).

**Figure 3:**
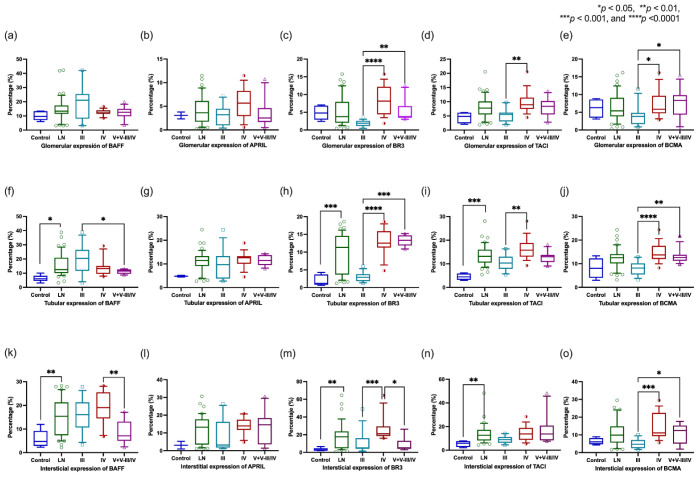
Association of BAFF system expression and proliferative LN classes according to glomerular, tubular, and interstitial zones. Glomerular expression of BR3 and BCMA was higher in class IV and V+V-III/IV groups than in class III; TACI showed a higher expression in class IV compared to class III (**c**–**e**). The tubular expression of BAFF was higher in the LN group, mainly in class III (**f**). The proliferative lesions (class IV) showed a higher expression of BR3, TACI, and BCMA (**h**–**j**). Compared to the interstitial BAFF system expression, BAFF, BR3, and TACI displayed higher expressions than the kidney controls (**k**). The Class IV group had higher expressions of BAFF, BR3, and BCMA (**k**,**m**,**o**). (**a**,**b**,**g**,**l**,**n**) did not show a statistical difference (*p* > 0.05). The *p*-value was obtained through Kruskal–Wallis and post hoc tests. Data are shown as median with IQR. BAFF: B-cells activating factor, APRIL: A proliferation-inducing ligand, BR3: BAFF receptor, TACI: Transmembrane activator and CALM interactor, BCMA: B-cell maturation antigen. IQR: Interquartile range, LN: lupus nephritis.

After the overall analysis, the LN tissues were stratified according to the ISN/RPS 2003 classification, and BAFF system expression was evaluated in three zones: glomerulus, tubule, and interstitium. At the glomerular level, both ligands and the three BAFF receptors exhibited similar expression between the renal control and LN classes (Figure 3a–e). However, the classes of LN demonstrated different receptor pattern expressions, showing higher expressions of BR3, TACI, and BCMA in classes IV (Figure 3c–e).

Similarly, the tubular zone was analyzed. BAFF, BR3, and TACI showed a higher percentage of expression than the renal controls, with statistical significance (Figure 3f,h,i). On the other hand, the tubular zone commonly expressed more APRIL and BCMA; however, no statistical significance was found (Figure 3g,j). When comparing BAFF system expression according to LN classes, classes IV and V+III/IV nephritis exhibited greater BR3, TACI, and BCMA expressions than the focal proliferative class (Figure 3h–j). In contrast, class III presented a higher expression of BAFF for another kind of nephritis (Figure 3a).

Additionally, expression analysis was performed in the interstitial zone. BAFF, BR3, and TACI exhibited higher expressions in LN compared to the control with statistical significance (Figure 3k,m,n). Otherwise, APRIL and BCMA showed a trend toward higher expression than the kidney controls; however, no statistical significance was found (Figure 3l,o). In the LN classes sub-analysis, BAFF, BR3, and BCMA expressions were higher in diffuse proliferative nephritis than in focal and membranous lesions (Figure 3k,m,o). On the other hand, TACI and APRIL did not show statistical significance (Figure 3l,n).

### 3.3. Correlation of Renal BAFF System Expression

**Figure 4:**
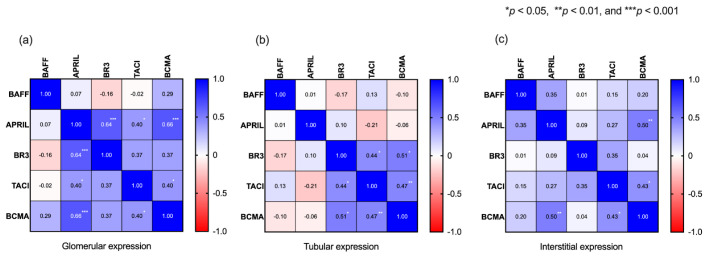
Matrix correlations of BAFF system expression according to zones. At the glomerular level, APRIL showed a positive correlation between all BAFF system receptors, and TACI correlated with BCMA (**a**). On the other hand, BR3, TACI, and BCMA showed a positive correlation at the tubular level (**b**). BCMA expression in the interstitium had a positive correlation with APRIL and TACI (**c**). The *p*-value was obtained through a Spearman rank correlation test. BAFF: B-cells activating factor, APRIL: A proliferation-inducing ligand, BR3: BAFF receptor, TACI: Transmembrane activator and CALM interactor, BCMA: B-cell maturation antigen.

Subsequently, a correlation analysis of BAFF system expression was performed. At the glomerular level (Figure 4a), APRIL displayed positive correlations with BR3 (r^2^ = 0.64), TACI (r^2^ = 0.40), and BCMA (r^2^ = 0.66), with statistical significance (*p* < 0.05). Additionally, TACI and BCMA correlated positively (r^2^ = 0.40, *p* < 0.05, Figure 4b). On the other hand, the tubular expression (Figure 4b) of BR3, TACI, and BCMA showed a positive correlation [BR3/TACI (r^2^ = 0.44), BR3/BCMA (r^2^ = 0.51), and TACI/BCMA (r^2^ = 0.47), *p* < 0.05]. Additionally, analysis at the interstitial level (Figure 4c) demonstrated a positive correlation between APRIL and BCMA (r^2^ = 0.50), as well as between BCMA and TACI (r^2^ = 0.43), with statistical significance (*p* < 0.05).

## 4. Discussion

LN is the most frequent life-threatening complication among African and Latin American SLE patients. LN is considered a predictor of flare disease [31] and causes end-stage renal disease (ESRD) in 10–30% of cases [32]. Multiple conventional and new renal biomarkers have been associated with LN [26]. However, a kidney biopsy remains the diagnostic gold standard and guides treatment according to the histological findings.

This study associated the histopathological activity index and wire-loop lesions with LN-IV. The most frequent histopathological features were a full-house phenomenon, tubular tumefaction, tubular atrophy, and tubulointerstitial fibrosis. However, these typical pathological features for the diagnosis of LN had a sensitivity ranging from 68 to 80%, with a specificity of 80–96% [33]. On the other hand, fibrinoid necrosis, fibrous crescents, interstitial fibrosis, and tubular atrophy were associated with poor renal prognosis and ERSD [34]. Thus, searching for potential biomarkers for the diagnosis and treatment of LN is imperative.

BAFF system imbalance has been associated with LN and SLE flare [22,24]. BAFF, APRIL, and mRNA expression of these ligands have been identified at the urinary level in LN patients [35,36]. However, the serum and urinary concentrations did not show a correlation [35], and their links have not been elucidated. Thus, what is/are the sources of BAFF/APRIL at the urinary level?

Possible answers for the detection of both ligands at the urinary level include proteinuria associated with the loss of glomerular membrane integrity secondary to immune complex deposition. Additionally, the source of BAFF system ligands could be related to infiltrating immune cells and/or the local production of resident renal cells. This study identified the in situ expression of BAFF, APRIL, and their receptors in glomerular and tubular zones, as well as in inflammatory cells at the interstitial level in proliferative LN tissues, presenting similar findings to those reported by Suso et al. [27].

In murine models with LN, immune complex deposition induces the recruitment of immune cells, favoring BAFF secretion and altering the position of renal T cells. These events promote the formation of tertiary lymphoid structures [37]. Despite local BAFF overproduction, maintaining lymphoid structures requires chemokine CXCL13. In murine models, CXCL13 is secreted by podocytes in LN [38]; this chemokine has been associated with kidney graft rejection [39] and postulated as an allograft rejection biomarker [40]. However, this chemokine was not evaluated in the present study.

In addition to the role of podocytes related to murine nephritis, tubular epithelial cells could be involved in the BAFF system. All members of this system were expressed in the tubular epithelium in our study, showing a positive correlation between BR3, TACI, and BCMA receptors. Schwarting et al. associated tubular epithelial cells with ectopic BAFF overproduction and the histopathological activity index in LN. Additionally, an autocrine loop phenomenon was documented through in vitro assays [41]. For BAFF receptors, adipocytes, keratinocytes, and microglia, there are no immune cells with ectopic expression [42,43,44]. In LN murine models, the tubular cells showed mRNA and tissular expression of BAFF receptors [41]. Hence, resident cells could show an ectopic expression of these receptors and amplify local inflammation in patients with LN.

Belimumab, a monoclonal antibody with BAFF targeting, is the only biological treatment approved to treat non-renal clinical domains in SLE [45]. In addition to conventional treatment in LN, Belimumab increases the probability of completing renal remission with a similar rate of adverse events [46,47]. Even in refractory LN, adding Belimumab to the cyclophosphamide and rituximab scheme could improve the renal response with an adequate security profile [48]. The BAFF system expression in resident renal cells and inflammatory infiltrating cells described in this study could support the use of anti-BAFF treatment. Additionally, TACI deletion in murine models is associated with the protection of LN [16].

The main limitations of this study are a reduced number of patients, only the inclusion of proliferative nephritis, the study design, a lack of clinical data, and the absence of labeling of B cells and CXCL13. The information obtained could help generate new therapeutic and diagnostic targets in LN.

## 5. Conclusions

The expression of BAFF and its receptors is mainly associated with LN class IV, and both inflammatory cells and resident kidney cells are involved in the expression of this system. Together, our results emphasize BAFF system participation as an important pathogenic factor for kidney involvement in SLE patients. However, these results should be taken with caution.

## Figures and Tables

**Table 1:** Histopathological features of proliferative LN tissues.

	Class III (*n* = 5)	Class IV (*n* = 6)	Class V and V+III/IV (*n* = 5)	Total (*n* = 16)	*p*-Value
Glomerulus, (IQR)	23 (14-36)	27 (15-33)	30 (19-42)	25 (18-34)	0.837
Histopathological activity index, (IQR)	5 (5-6)	15 (12-16)	5 (4-6)	6 (5-14)	0.001
Histopathological chronicity index, (IQR)	4 (3-4)	3 (3-3)	3 (2-3)	3 (2-3)	0.239
Full-house phenomenon, (%)	4 (80)	6 (100)	5 (100)	15 (94)	0.279
Crescents, (%)	2 (40)	6 (100)	2 (40)	10 (62.5)	0.056
Glomerular sclerosis, (%)	4 (80)	4 (67)	4 (80)	12 (75)	0.356
Wire-loop lesions, (%)	0	5 (83)	0	5 (31)	0.002
Karyorrhexis, (%)	3 (60)	6 (100)	3 (60)	12 (75)	0.202
Neutrophilic infiltrated, (%)	3 (60)	6 (100)	3 (60)	12 (75)	0.202
Hyaline thrombus, (%)	1 (20)	3 (50)	0	4 (25)	0.155
Tubular atrophy, (%)	4 (80)	6 (100)	5 (100)	14 (88)	0.504
Tubular tumefaction, (%)	4 (80)	6 (100)	5 (100)	15 (94)	0.309
Tubular-interstitial infiltrated, (%)	5 (100)	4 (67)	3 (60)	12 (75)	0.288
Tubulointerstitial fibrosis, (%)	4 (80)	5 (83)	4 (80)	13 (81)	0.986

Data are shown as the median, IQR, frequencies, and percentages, as appropriate. The *p*-value was obtained using Fisher’s test or Kruskal–Wallis and Dunn’s post hoc test, as appropriate. LN: lupus nephritis, IQR: interquartile range.

## Data Availability

Not applicable.
